# A Novel Algorithm Using Cell Population Data (VCS Parameters) as a Screening Discriminant between Alpha and Beta Thalassemia Traits

**DOI:** 10.3390/diagnostics11112163

**Published:** 2021-11-22

**Authors:** Angeli Ambayya, Santina Sahibon, Thoo Wei Yang, Qian-Yun Zhang, Rosline Hassan, Jameela Sathar

**Affiliations:** 1 Haematology Department, Hospital Ampang, Ampang 68000, Selangor, Malaysia; jsathar@hotmail.com; 2 Department Haematology, School of Medical Sciences, Universiti Sains Malaysia, Kota Bharu 15200, Kelantan, Malaysia; roslin@usm.my; 3 Gribbles Pathology Malaysia, Petaling Jaya 46100, Selangor, Malaysia; santina@gribbles.com.my; 4 Straits Scientific Malaysia, Ampang 68000, Selangor, Malaysia; jimmy@3s.com.my; 5 Department of Pathology, University of New Mexico, Albuquerque, NM 87131, USA; qzhang@salud.unm.edu

**Keywords:** cell population data, algorithm, α-thalassemia, β-thalassaemia, VCS parameters

## Abstract

Thalassemia is one of the major inherited haematological disorders in the Southeast Asia region. This study explored the potential utility of red blood cell (RBC) parameters and reticulocyte cell population data (CPD) parameters in the differential diagnosis of α and β-thalassaemia traits as a rapid and cost-effective tool for screening of thalassemia traits. In this study, a total of 1597 subjects (1394 apparently healthy subjects, 155 subjects with α-thalassaemia trait, and 48 subjects with β-thalassaemia trait) were accrued. The parameters studied were the RBC parameters and reticulocyte CPD parameters derived from Unicel DxH800. A novel algorithm named αβ-algorithm was developed: (MN-LMALS-RET × RDW) − MCH) to discriminate α from β-thalassaemia trait with a cut-off value of 1742.5 [AUC = 0.966, sensitivity = 92%, specificity = 90%, 95% CI = 0.94–0.99]. Two prospective studies were carried: an in-house cohort to assess the specificity of this algorithm in 310 samples comprising various RBC disorders and in an interlaboratory cohort of 65 α-thalassemia trait, and 30 β-thalassaemia trait subjects to assess the reproducibility of the findings. We propose the αβ-algorithm to serve as a rapid, inexpensive surrogate evaluation tool of α and β-thalassaemia in the population screening of thalassemia traits in geographic regions with a high burden of these inherited blood disorders.

## 1. Introduction

Thalassaemia and haemoglobin variant disorders occur widely worldwide, with an estimation of 7% of the world population being carriers, as reported by the World Health Organisation (WHO) [1]. About 300,000–500,000 children were diagnosed with significant haemoglobin disorders, and of these, about 80% were born in developing countries [1]. In Malaysia, thalassaemia is the commonest single gene disorder characterised by defects in synthesising one or more globin chains [2]. Individuals with homozygous and double heterozygous mutations are associated with thalassaemia major or intermedia phenotype, high morbidity and mortality, whereas individuals with heterozygous mutations are carriers without exhibiting adverse morbidity [2]. Alpha (α) and beta (β) thalassaemia are the commonest types of thalassaemia in Malaysia [3].

Malaysian Thalassaemia Registry (2009) revealed that 3310 out of 4541 registered patients consist of transfusion-dependent β-thalassaemia major and HbE/β-thalassaemia patients [4]. Clinical management of these patients includes transfusion, iron chelation therapy, and haematopoietic stem cell transplantations, which are expensive and a burden to the country’s healthcare system [5]. Besides patient management, the National Thalassaemia Prevention and Control Programme was actively carried out nationwide to provide population screening and counselling at primary healthcare facilities and health education and promotions to disseminate knowledge and awareness about the consequences of these disorders [4].

α-thalassaemia is characterised by the deficiency or absence of α-globin chain synthesis due to deletions of one or more α-globin genes located in chromosome 16 [6]. The severity of α-thalassaemia is characterised by the number of gene deletions, where single gene deletions result in α-thalassaemia silent carriers, two gene deletions ensue α-thalassaemia trait (minor), three gene deletions eventuate in haemoglobin H (HbH), and four-gene deletions result in haemoglobin Bart’s, which usually results in fatal hydrops fetalis that have very high fatality rate [7]. On the other hand, β-thalassaemia is caused by the reduced or absent β-globin chain synthesis due to mutations in the β-globin gene on chromosome 11 [8]. Generally, β-thalassaemia is categorised as β-thalassaemia trait (minor), β-thalassaemia intermedia, and β-thalassaemia major based gene defects and severity of the decrease in β-globin chains production [9].

Preliminary identification of thalassaemia carriers is carried out by a screening program by full blood count (FBC) analysis as the baseline test to scrutinise the red blood cell indices, followed by morphologic examination of peripheral blood smears and subsequent confirmation by high-performance liquid chromatography (HPLC), haemoglobin (Hb) electrophoresis, and molecular genetics testing [4]. Each of the confirmation tests mentioned has its limitation, necessitating a combination of a few tests for differential diagnosis of α and β-thalassaemia. For instance, HPLC is not sufficient to discreetly detect variants as it is not sensitive and specific enough for an α-thalassaemia detection, especially in the presence of haemoglobin Constant Spring (non-deletion α-thalassaemia), indicated by the presence of a very tiny peak of Hb that is often missed [10,11]. In contrast, it is seen on the capillary electrophoresis (CE) electropherogram. Besides that, Hb electrophoresis also cannot be used as the sole technique to distinguish α- and β-thalassaemia. Haemoglobin electrophoresis with β-thalassaemia trait usually shows reduced or absent adult haemoglobin (HbA), elevated levels of haemoglobin A2 (HbA2), and increased foetal haemoglobin (HbF) [10,12]. However, a normal concentration of HbA2 does not rule out the β-thalassaemia trait, especially if there is a concurrent iron deficiency or δ-thalassaemia, which can lower HbA2 levels into the normal range. Therefore, a combination of other methods is required for confirmatory diagnosis [12]. Combinations of these multiple testing are rather costly and require expertise to make accurate and reliable diagnoses as there are substantial overlaps among these disorders [10]. Numerous combinations of red cell indices have been suggested to distinguish different types of thalassaemia as this would allow cost optimisation as only patients requiring further investigations are subjected to expensive haemoglobin electrophoresis and molecular genetics testing.

Along with the advent of new generation full blood count (FBC) analysers, advanced parameters such as cell population data (CPD), which measure the characteristics of cells based on multiple light scatters, are being explored to understand the diagnostic utilities of these parameters better. The CPD parameters provide information on the volume (V), conductivity (C), and light scatter angles (ALL, LALS, LMALS, UMALS, and MALS) for reticulocytes, which are important in the assessment of bone marrow red blood cell production efficiency and detection of haemoglobin disorders such as thalassaemia and anaemia [13]. In this study, we explored the potential utility of reticulocyte CPD and aimed to construct an algorithm that could serve as a rapid tool and an inexpensive surrogate method for the discrimination of α- and β-thalassaemia traits in population-based thalassemia screening programs held not only in Malaysia but also in other countries with a high burden of these red cell disorders.

## 2. Materials and Methods

A total of 1597 subjects were included in the primary assessment and algorithm development cohort. The subjects were recruited between September 2011 and February 2019 in a study to establish reference intervals for haematological parameters in Malaysian adults reported in Ambayya et al. (2014) [14]. These cases were retrospectively analysed and categorised into 1394 apparently healthy subjects, 155 subjects with α-thalassemia trait, and 48 subjects with β-thalassemia trait.

Two ethical approvals were obtained for this study from the Medical Research Ethics Committee of the Ministry of Health Malaysia. First, for the recruitments of subjects in the reference interval establishments for haematological parameters (Research ID 10-277-5480) and second, for the exploration of haematological parameters available in FBC analysers in haematological disorders (NMRR 17-2708-38327). Written informed consent was obtained from all subjects before recruitment. Samples were processed according to the recommendation by International Council for Standardisation in Haematology [15,16,17].

All samples were processed as described in Ambayya et al. (2014) [14]. Initial screening test to rule out anaemia and thalassemia include full blood count (FBC) analysis of red cell indices (MCH < 27 pg, MCV < 80 fl, RDW > 14%) that was performed on Unicel DxH 800 (Beckman Coulter, Miami, FL, USA). Then, peripheral blood film (PBF) was processed using SP1000i automated slide maker (Sysmex, Kobe, Japan) and reviewed by qualified laboratory personnel. Haemoglobin analysis was carried out on Capillarys 2 (Sebia, France) and followed by H inclusion detected by supravital brilliant cresyl blue (BCB) staining (Merck Millipore, Darmstadt, Germany). Iron studies assayed in this study include serum ferritin (Modular E170, Roche, Switzerland) and soluble transferrin receptor (Cobas Integra 400, Roche, Switzerland). In subjects with increased serum ferritin [males > 400 ng/mL, females > 150 ng/mL], C-reactive protein was performed using AU480 (Bekman Coulter, USA). DNA testing for confirmation of α -thalassaemia carriers was conducted in cases selected cases that required definitive diagnosis. The analyses’ pipeline adopted in this study was based on Malaysia’s Management of transfusion-dependent thalassaemia (Clinical Practice guideline) v2009 [4].

Comparison of red blood cell (RBC) and reticulocyte routinely reported parameters, research parameters, and cell population data (CPD) were retrieved from Unicel DxH 800. Description of the CPD parameters is in Supplementary S1. Several strategies were developed first to scrutinise these parameters between the control group (apparently healthy individuals) and subjects diagnosed with α and β-thalassaemia and then select significant indices for a mathematical algorithm development that displayed the largest area under the curve (AUC) as a single parameter.

All statistical analyses were performed using IBM SPSS Statistics 22 software (SPSS, Chicago, IL, USA). The Kolmogorov-Smirnov test of normality was carried out for distribution assessment of each parameter (RBC parameters, research parameters, and CPD). One-way variance analysis (ANOVA) with homogeneity of variance test was conducted and followed by a post-hoc test. Tukey test was done on parameters that fulfilled the homogeneity of variance, while Games-Howell test for the parameters that did not fulfil the homogeneity of variance. Then, the Welch test was done to verify parameters that did not fulfil the homogeneity of variance. For the initial assessment of differences between the α and β-thalassaemia trait subjects, an independent t-test was conducted to test the parameters at a significance level of 0.05. Receiver Operating Characteristic (ROC) curves were generated for parameters that yielded *p*-values of <0.05. ROC analysis with *p*-values of <0.05 and AUC > 0.8 was considered significant in this study, and for each shortlisted parameter, cut-off points were determined by considering the sensitivity and specificity.

A novel algorithm, termed as αβ-algorithm, was formed by utilising routinely reported RBC parameters and reticulocyte CPD with AUC > 0.9 to discriminate α and β-thalassaemia traits. This algorithm was tested in comparison with a control group cohort (apparently healthy individuals), α and β-thalassaemia traits. Following that, two prospective validation cohorts were designed and carried out to ensure the reliability, reproducibility, and robustness of (αβ-algorithm) when heterogenous RBC disorders are present in a diagnostic setting: an in-house cohort and an interlaboratory cohort in collaboration with Gribbles Pathology, Malaysia. For the in-house validation cohort, 310 samples were recruited from various red cell disorders subjects (119 α-thalassemia trait, 48 β-thalassemia trait, 15 haemoglobin E trait, 84 iron deficiency anaemia (IDA), and 44 iron deficiency (ID)). We compared the distribution of cases among these red cell disorders to assess the overlap of cut-offs between other cases (non-α or β-thalassemia trait). As for the interlaboratory cohort, 95 samples were included (65 α-thalassemia trait, 30 β-thalassemia trait) recruited during general health screening programs. We used VassarStats clinical calculator (http://vassarstats.net/clin1.html, accessed on 16 September 2021) to obtain sensitivity, specificity, positive, and negative predictive values to discriminate α and β-thalassaemia traits [15].

## 3. Results

### 3.1. Development of (αβ Algorithm) Using a Primary Retrospective Cohort

A total of 1597 subjects (1394 control group/apparently healthy; 155 α-thalassemia trait; 48 β-thalassemia trait) were assessed in this study. The distribution of cases and summary of statistics for RBC parameters, sTFR, serum ferritin, and haemoglobin analysis (HBA, HBA2, and HBF) are detailed in Table 1. After performing normality testing, we then compared the groups (control, α, and β-thalassaemia traits) by performing ANOVA and post hoc tests, and the diagnostic performance was determined by ROC analyses, as summarised in Supplementary S2.

**Figure 1:**
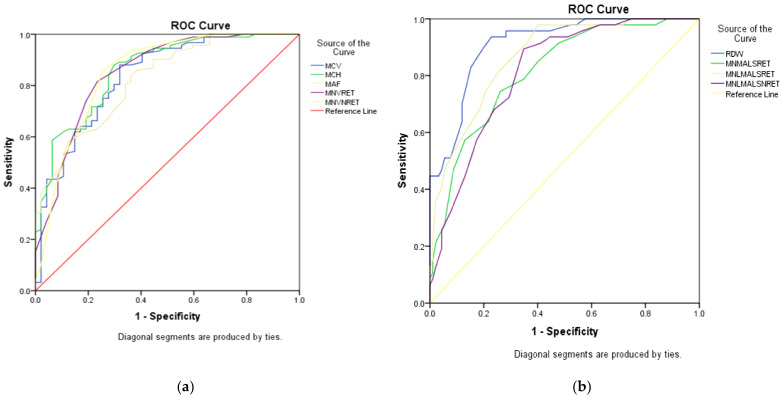
(**a**,**b**) ROC curves of RBC parameters and reticulocyte CPD to distinguish α from β thalassaemia trait.

**Figure 2:**
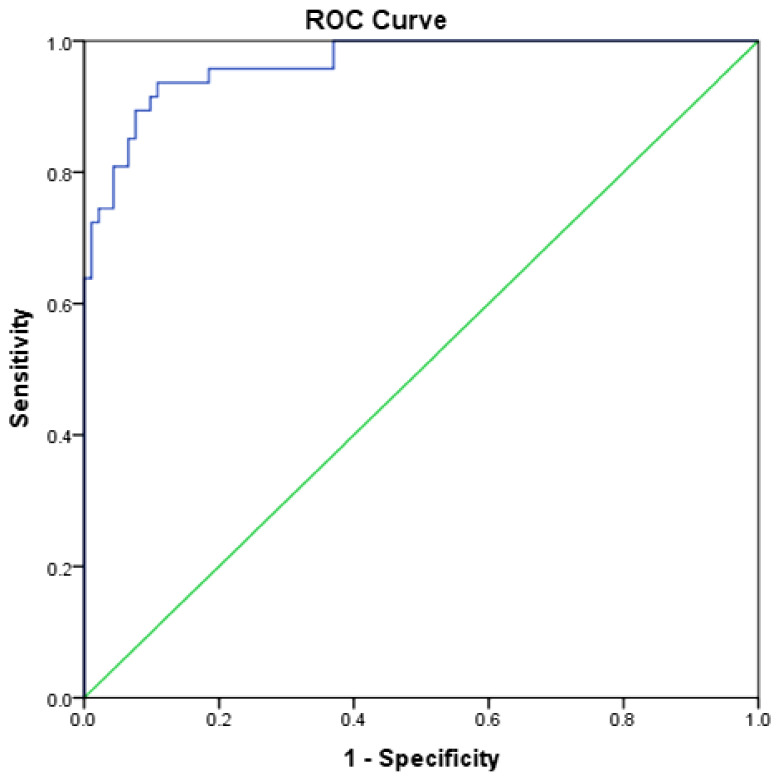
The ROC curve for discriminating α from β-thalassaemia traits generated from the αβ-algorithm.

**Figure 3:**
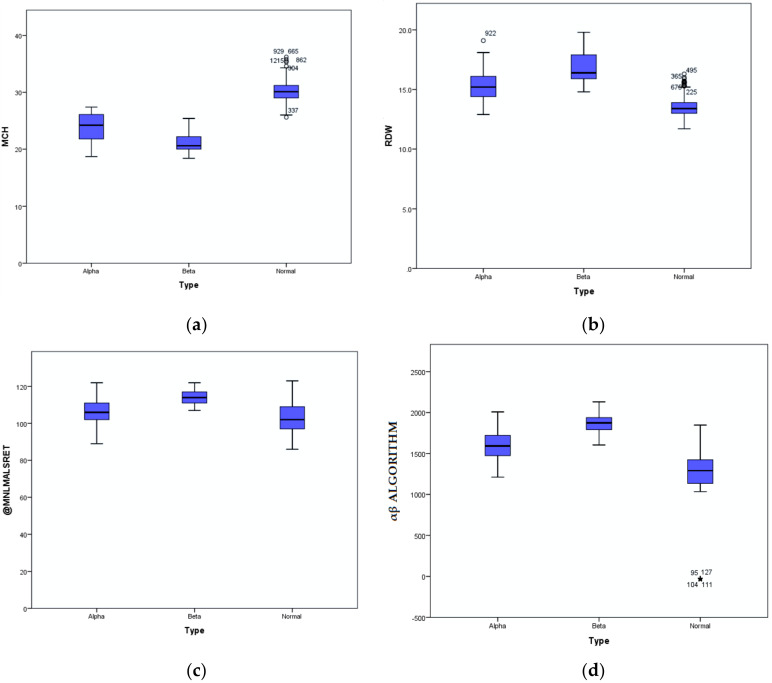
(**a**–**d**) Box and whisker plot for MCH, RDW, MN-LMALS-RET (labelled as @MNLMALSRET in Figure 3c and the αβ-algorithm between the α and β-thalassaemia trait and the control group (apparently healthy subjects). * refers to the outliers.

**Figure 4:**
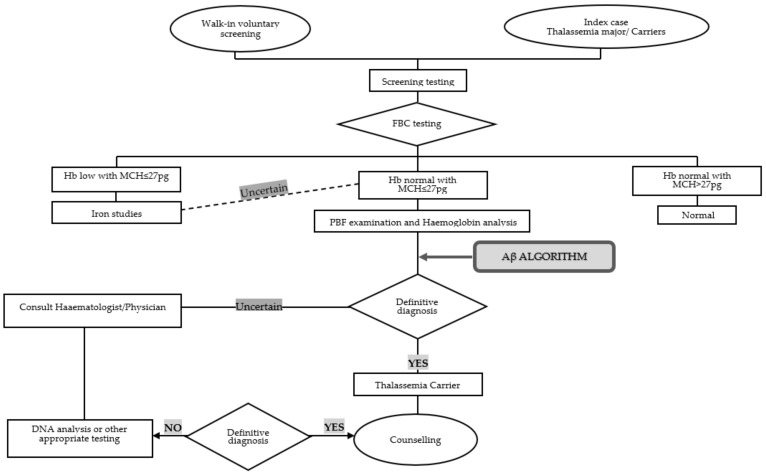
Flowchart representing the αβ-algorithm incorporated into the screening algorithm for voluntary and cascade screening in Malaysia’s Clinical Practice Guideline for Management of Transfusion Dependent Thalassaemia [4].

ROC analysis involved 203 subjects (155 α-thalassaemia trait and 48 β-thalassaemia trait), in which five parameters were notably higher (MCV, MCH, MAF, MN-V-RET, MN-V-NRET) in α than in the β-thalassaemia trait. The following parameters were lower in α than in the β-thalassaemia trait: RDW, MN-MALS-RET, MN-LMALS-RET, and MN-LMALS-NRET, as shown in Table 2. Three parameters (MN-LMALS-RET, MCH, and RDW) possessed the largest AUC distinguishing α from β-thalassaemia. To improve the sensitivity and specificity of these parameters, a mathematical algorithm (αβ-algorithm) was devised (MN-LMALS-RET × RDW) − MCH) to robustly distinguish α from β-thalassaemia trait with a cut-off of 1742.5, the AUC, sensitivity, and specificity were 0.966 (95% CI: 0.94, 0.99), 92% and 90%, respectively. ROC curves of RBC parameters and reticulocyte CPDs are depicted in Figure 1a,b. Figure 2 displays the ROC curve for the αβ-algorithm. Box and whisker plots for the for MCH, RDW, MN-LMALS-RET, and the αβ-algorithm that delineate the α,β-thalassaemia trait and the control group (apparently healthy subjects) are represented in Figure 3a–d. The αβ-algorithm is incorporated into the screening algorithm for voluntary and cascade screening in the Clinical Practice Guideline for Management of Transfusion Dependent Thalassaemia in Malaysia, as depicted in Figure 4 [4].

### 3.2. αβ-Algorithm Validation Studies

#### 3.2.1. In-House Validation

**Figure 5:**
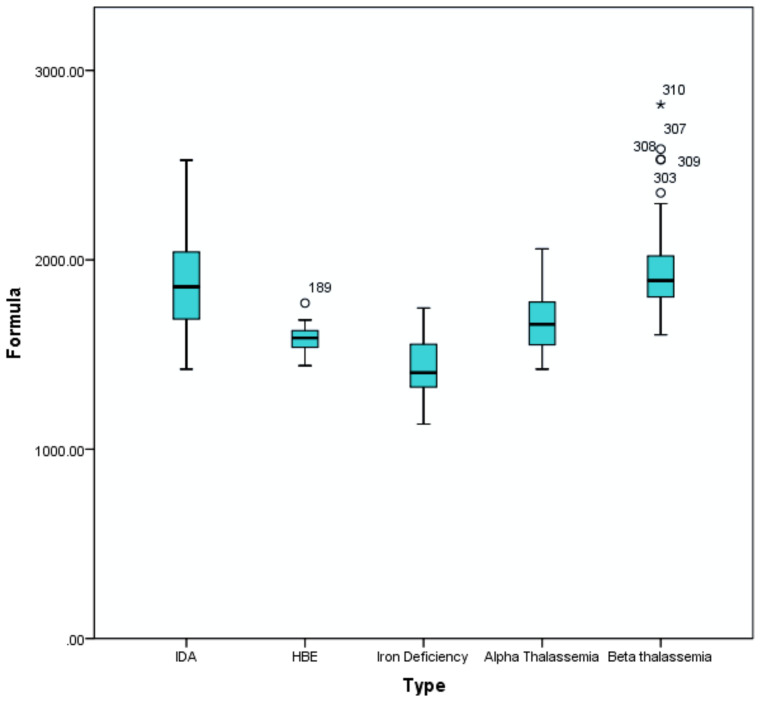
Box and whisker plot for the αβ-algorithm in various red cell disorders (IDA, HbE trait (labelled as HBE in this figure), iron deficiency, α thalassaemia trait (labelled as alpha thalassemia), and β-thalassaemia trait (labelled as beta-thalassemia). Outliers are marked as “*” and “⁰” in the HbE trait and β-thalassaemia trait groups.

In-house validation in a cohort of 310 subjects comprising various red cell disorders as summarised in Table 3 and Supplementary S3 and corresponding box and whisker plot is depicted in Figure 5. As shown, between the quartile 1 (Q1) to quartile 3 (Q3) of α thalassemia and β thalassemia traits, there was no significant overlap, comprising the majority of the cases included in this cohort. The overlapping cases require confirmation by HPLC and DNA testing. There is a significant overlap between the IDA and β thalassemia trait.

#### 3.2.2. Interlaboratory Validation Cohort

An interlaboratory validation cohort that included 95 samples (65 α-thalassemia trait, 30 β-thalassemia trait) were subjected to the developed mathematical algorithm. β-thalassaemia trait was assigned as the positive group, and α-thalassaemia trait was assigned as the negative group, as shown in Table 4 and Supplementary S4. This αβ-algorithm yielded a sensitivity of 92.85% (CI:0.75, 0.99) and specificity of 94.03% (CI:0.84, 0.98) in discriminating β-thalassaemia trait from the α-thalassaemia trait with a positive predictive accuracy of 86.67% (CI:0.68, 0.95) and negative predictive accuracy of 96.9% (CI:0.88, 0.99).

## 4. Discussion

Distinguishing between various thalassaemia traits is essential in clinical decision-making as it will influence the treatment options and outcome of the patients [16]. However, the diagnosis of thalassaemia traits requires time and resources as various screening and confirmatory testing, including FBC, morphological review of blood smears, haemoglobin electrophoresis, and molecular genetic analysis to establish a reliable and robust diagnosis [17]. Several algorithms based on RBC indices have been proposed to differentiate iron deficiency anaemia and thalassaemia traits [17], including Mentzer Index [19], England & Fraser Index [20], Shine and Lal formula [21], Ehsani formula [22], Srivastava formula [23], Palestinian population [24], Green & King Index [25], and RDW Index [26].

In more recent studies, researchers have been exploring machine learning algorithm-based studies with advancements in data science [27,28]. In a study in Thailand, a web-based prediction tool for discrimination of thalassemia trait and IDA was developed using a machine learning algorithm. However, in this study, the authors did not delineate the subtypes of thalassemia but created a support vector machine (SVM) model to distinguish IDA from thal trait, named ThalPred [27]. One of the largest studies performed in Israel validated Shine’s formula and in-house developed SVM formula. This study included a total of 64,586 subjects. Their SVM formula displayed high sensitivity(>98%) and >99.77% negative predictive value that is robust in distinguishing the β-thalassemia carrier from normal count subjects and iron-deficient women [28].

Previously, using the CPD parameters generated by Beckman Coulter DxH 800, Ng and the team proposed an algorithm to differentiate IDA from thalassaemia traits among its subjects. With a cut-off value of 23, the area under the curve (AUC) of 0.995 (95% CI of 0.99–1.00), the algorithm achieved a sensitivity of 97% and specificity of 99.1%. They suggested that no biochemical marker of iron status such as serum ferritin testing is required with this formula; hence, simplified diagnostic workup of IDA and thalassemia was proposed [16]. However, these formulas were devised from various populations with varying sensitivity and specificity, with none specific for differentiation of α and β-thalassaemia, without incorporating reticulocyte CPD parameters, as done in this present study [17,29,30].

The differentiation of α and β-thalassaemia based on the red blood cells parameter (Hb level, MCH, MCV, and RBC level) was performed in 2018 by R. Azma et al. in an LH 750 FBC analyser by Beckman Coulter (USA). A total of 299 subjects (α-thalassaemia traits, n = 160 and β-thalassaemia traits, n = 139) were included for this study. It showed that α-thalassaemia carriers have higher Hb level (α-thalassaemia carrier: 12.1 ± 1.31; β-thalassaemia carrier: 10.6 ± 1.8, *p*-value: 0.00 *), MCV (α-thalassaemia carrier: 71.0 ± 6.1; β-thalassaemia carrier: 68.7 ± 7.2, *p*-value: 0.044 *), MCH (α-thalassaemia carrier: 22.8 ± 2.3; β-thalassaemia carrier: 21.8 ± 2.7, *p*-value: 0.056) RBC level (α-thalassaemia carrier: 5.3 ± 0.7; β-thalassaemia carrier: 4.9 ± 0.9, *p*-value: 0.001 *), compared to β-thalassaemia carriers [31]. Although this study was not done based on CPD parameters, similar results were observed in our present study. The MCV and MCH parameters are shown to have notably higher values among subjects with α-thalassaemia trait compared to β-thalassaemia trait, in accordance with the findings from this study, as depicted in Table 1.

In this present study, we successfully developed an algorithm (αβ-algorithm) based on reticulocyte CPD parameters that will serve as the downstream tool after FBC is performed to distinguish α versus β-thalassaemia trait, using Beckman Coulter Unicel DxH 800. The ROC-curve analysis revealed three parameters with the highest AUC, MN-LMALS-RET, MCH, and RDW, with good sensitivity and specificity (Figure 1a,b and Figure 2). The αβ-algorithm was devised by combining the aforementioned parameters ((MN-LMALS-RET × RDW) − MCH) that resulted in higher AUC with better sensitivity and specificity to discriminate α from β-thalassaemia traits. The rationales for the αβ-algorithm were MN-LMALS-RET, which measures the red blood cell volume in the reticulocyte channel through lower median angle light scattering [32], MCH indicates the amount of haemoglobin in a red blood cell [33], and RDW helps to measure variation in red blood cell size [12].

Our prospective study revealed the usefulness of the αβ-algorithm as a downstream pipeline following FBC analysis, as depicted in Figure 4. Based on Figure 5, when this algorithm was evaluated in a cohort of various red cell disorders, there was an overlap between the subgroups, especially between the β-thalassemia trait and IDA groups. Nevertheless, the diagnostic pipeline for cases suspected as IDA differs in the Malaysian clinical practice guideline, so this overlap will not impact the role of this algorithm in the differential diagnosis of α and β-thalassemia. Interlaboratory validation showed that the findings are reproducible in another laboratory setting with similar Unicel DxH 800, hence reiterating the usefulness of the αβ-algorithm in the screening of thalassemia cases, in particular, α and β-thalassemia in geographical locations with a high prevalence of thalassaemias such as Malaysia and other Asian countries.

There are several limitations of this study: First, it included a relatively low number of α and β-thalassemia cases. Secondly, β-thalassemia group, the maximum value of MCV was 82 that overlaps with the minimum MCV of 80 in the healthy control group. In such cases, other RBC indices (RBC, MCH) and HPLC would aid in the differential diagnosis of β-thalassemia. Ideally, molecular analysis for both alpha and beta mutations needs to be performed on all samples so as not to miss out on the silent α or β thalassemia during screening. However, we did not perform molecular analysis on all cases because of the high testing cost and rarity of silent β-thalassemia [34,35].

We propose the vigorousness of this αβ-algorithm be validated in a larger cohort in other geographical locations that exhibit high prevalence and incidence of α and β-thalassaemia traits. Based on this study, we recommend the FBC analysis is performed within 6 h of sampling following International Council for Standardisation in Haematology (ICSH) guidelines [36]. Apart from that, the transportation and storage of samples condition must meet the guidelines recommended by The Clinical and Laboratory Standard Institute (CLSI) [37]. Any deviation from adhering strictly to these guidelines may affect the sensitivity and specificity of this algorithm as CPD parameters are highly affected by the structural changes of the cells.

## 5. Conclusions

Devising an algorithm that accurately distinguishes α and β-thalassaemia traits using CPD parameters derived from FBC analyser is essential in the pipeline of large population-based screening carried out in Malaysia as high prevalence of these inherited disorders are reported. Implementing the αβ-algorithm in the screening laboratory will promote cost optimisation as only suspected subjects will be included in the downstream pipeline before performing haemoglobin electrophoresis and/or further specific genetic testing, as depicted in Figure 4. The applicability of the αβ-algorithm is relatively straightforward without involving any additional cost to the diagnosis and requires no sophisticated analysis and expertise. Hence, we strongly recommend adopting this αβ-algorithm in all screening laboratories with access to similar CPD parameters nationwide and other geographical regions with a high prevalence of thalassaemia-related disorders [38,39].

## Figures and Tables

**Table 1:** Distribution of cases and statistics of RBC parameters, serum ferritin, haemoglobin analysis (HBA, HBA2, and HBF).

Groups	Statistics	HB (g/dL)	RBC (10 /mcL)	HCT (%)	MCV (fL)	MCH (pg)	MCHC (g/dL)	RDW (%)	TSfR (mg/L)	Ferritin (mcg/L)	HBA (%)	HBA2 (%)	HBF (%)
**Alpha trait**	**N**	**155**	**155**	**155**	**155**	**155**	**155**	**155**	**155**	**155**	**155**	**155**	**155**
	Mean	12.56	5.15	39.03	76.27	24.57	32.19	15.43	4.14	117.03	97.50	2.43	0.58
	SD	1.22	5.08	3.46	5.66	2.18	0.86	1.45	1.39	138.70	0.36	0.25	0.53
	Med	12.45	0.59	38.60	77.60	25.10	32.10	15.30	3.90	72.00	97.60	2.40	0.35
	Min	10.10	3.57	32.20	60.10	19.20	29.90	12.90	1.50	3.00	95.50	1.90	0.20
	Max	16.30	6.76	49.60	98.50	32.80	35.30	21.40	9.90	766.00	98.10	3.50	2.20
	Range	6.20	3.19	17.40	38.40	13.60	5.40	8.50	8.40	763.00	2.60	1.60	2.00
**Beta trait**	**N**	**48**	**48**	**48**	**48**	**48**	**48**	**48**	**nd**	**nd**	**48**	**48**	**48**
	Mean	11.64	5.43	36.69	68.00	21.57	31.73	17.31	-	-	93.83	5.23	1.13
	SD	0.87	0.55	2.60	6.24	2.17	0.73	2.01	-	-	0.96	0.49	0.76
	Med	11.80	5.39	36.80	65.40	20.70	32.00	16.55	-	-	93.70	5.40	1.00
	Min	10.20	4.51	32.00	61.50	18.30	29.60	14.80	-	-	91.70	4.40	0.30
	Max	13.20	6.45	40.90	82.10	26.30	32.90	24.30	-	-	95.40	6.00	3.50
	Range	3.00	1.94	8.90	20.60	8.00	3.30	9.50	-	-	3.70	1.60	3.20
**Control group (healthy male)**	**N**	**477**	**477**	**477**	**1394 ^a^**	**1394 ^a^**	**1394 ^a^**	**1394 ^a^**	**1394 ^a^**	**477**	**1394 ^a^**	**1394 ^a^**	**1394 ^a^**
	Mean	14.91	4.83	43.84	89.61	30.12	33.62	13.51	3.19	265.56	97.34	2.61	0.58
	SD	1.06	0.41	2.73	4.00	1.58	0.87	0.72	0.90	230.02	0.32	0.24	0.42
	Med	14.90	4.91	43.60	89.50	30.10	33.60	13.40	3.10	217	97.40	2.60	0.40
	Min	12.50	3.83	37.40	80.30	26.9	30.10	11.70	0.90	37	94.90	1.10	0.20
	Max	16.90	5.61	48.20	107.80	43.50	48.20	19.00	8.80	1138	98.90	3.50	2.50
	Range	4.40	1.78	10.80	27.50	17.90	18.10	7.30	7.90	1101	4.00	2.40	2.30
**Control group (healthy female)**	**N**	**917**	**917**	**917**	**1394 ^a^**	**1394 ^a^**	**1394 ^a^**	**1394 ^a^**	**1394 ^a^**	**917**	**1394 ^a^**	**1394 ^a^**	**1394 ^a^**
	Mean	13.00	4.24	38.54	89.61	30.12	33.62	13.51	3.19	106.89	97.34	2.61	0.58
	SD	0.81	0.32	2.61	4.00	1.58	0.87	0.72	0.90	114.04	0.32	0.24	0.42
	Med	12.90	4.23	38.50	89.50	30.10	33.60	13.40	3.10	74	97.40	2.60	0.40
	Min	11.60	2.97	26.80	80.30	26.9	30.10	11.70	0.90	14	94.90	1.10	0.20
	Max	14.90	5.15	44.40	107.80	43.50	48.20	19.00	8.80	669	98.90	3.50	2.50
	Range	4.60	2.18	17.60	27.50	17.90	18.10	7.30	7.90	655	4.00	2.40	2.30

nd refers to no data. Serum ferritin and sTfR are not tested in β-thalassaemia trait subjects. **^a^** Based on the haematological reference range for Malaysian adults [18], specific gender-based calculations were performed for HB, RBC, HCT, and serum ferritin. Whereas for the other parameters (MCV, MCH, MCHC, RDW, TSfR, Ferritin, HBA, HBA2, and HBF), no gender-based calculation was done as no clinically gender-based associated variation was reported [18].

**Table 2:** The ROC data elaboration for each reticulocyte CPD to distinguish α from β-thalassaemia.

Parameters	AUC	95% CI	Cut-Off		Sens (%)	Spec (%)
			α-Thalassaemia	β-Thalassaemia		
αβ-algorithm	0.966	0.939, 0.993	<1742.50	>1742.50	91.49	90.22
RDW	0.911	0.864, 0.958	<15.85	>15.85	82.98	84.78
MN-LMALS-RET	0.868	0.810, 0.927	<109.50	>109.50	80.85	75.00
MCH	0.861	0.796, 0.925	>22.30	<22.30	76.09	74.47
MN-V-RET	0.853	0.783, 0.923	>47.50	<47.50	81.52	76.59
MN-V-NRET	0.853	0.780, 0.926	>35.50	<35.50	85.87	74.47
MCV	0.836	0.764, 0.909	>70.80	<70.80	75	74.47
MAF	0.823	0.753, 0.894	>8.95	<8.95	70.65	70.21
MN-MALS-RET	0.816	0.744, 0.888	<118.50	>118.50	74.47	73.91
MN-LMALS-NRET	0.813	0.743, 0.883	<43.50	>43.50	72.34	70.65

AUC, area under the curve; 95% CI, 95% confidence interval; thal, thalassaemia; MCV, mean corpuscular volume; MCH, mean corpuscular haemoglobin; MAF, microcytic anaemia factor; RDW, red blood cells distribution width; MN-LMALS-RET, lower median angle light scatter reticulocyte; Sens, sensitivity, Spec, specificity. Refer to Supplementary S1, Description of CPD Parameters for the CPD abbreviations).

**Table 3:** In-house validation of the αβ-algorithm to distinguish between various red cell disorders.

Algorithm	Alpha Trait	Beta Trait	HbE Trait	IDA	ID
N	119	48	15	84	44
Mean	1667.82	1960.19	1586.31	1874.90	1430.13
Med	1660.10	1890.40	1587.60	1857.90	1403.65
SD	148.21	248.65	80.58	245.80	153.62
Max	2058.20	2819.50	1771.80	2526.20	1745.80
Min	1422.70	1604.30	1441.20	1422.80	1132.90
Variance	21,966.37	61,829.15	6493.59	60,417.72	23,598.58

**Table 4:** Interlaboratory comparison of the αβ-algorithm in differentiating α-thalassemia trait and 30 β-thalassemia trait.

	Condition			
Algorithm	Prediction	Absent	Present	Total
(MNLMALSRET × RDW) − MCH	Positive	4	26	30
	Negative	63	2	65
	Total	67	28	95

## Data Availability

The data used in this study are available as Supplementary Materials (Supplementary S1–S4).

## Supplementary Materials

The following are available online at https://www.mdpi.com/article/10.3390/diagnostics11112163/s1. Supplementary Materials are available as the following files: Supplementary S1 Description of CPD Parameters.docx, Supplementary S2 ROC analysis.xlsx, Supplementary S3 in-house cohort.xlsx, and Supplementary S4 Interlaboratory Cohort.xls.
