# Supplementary material for: A Novel Algorithm Using Cell Population Data (VCS Parameters) as a Screening Discriminant between Alpha and Beta Thalassemia Traits
Source: Diagnostics (Basel). 2021 Nov 22;11(11):2163. doi: 10.3390/diagnostics11112163 (PMC8619269; doi:10.3390/diagnostics11112163)
Supplement: Supplementary file 1 [file diagnostics-11-02163-s001.zip › diagnostics-1443553-supplementary/diagnostics-1443553-supplementary/Supplementary/Supplementary 1 Description of CPD Parameters.pdf]

1. Cell population data (CPD) for reticulocyte related parameters available in Unicel DxH800 full blood count analyser by Beckman Coulter

Parameter	Mean Retic	SD Retic	Mean Non-Retic	SD Non-Retic
**Volume**	@MN-V-RET	@SD-V-RET	@MN-V-NRET	@SD-V-NRET
**Conductivity**	@MN-C- RET	@ SD -C- RET	@MN-C- NRET	@ SD -C- NRET
**Upper Median Angle Light Scatter (UMALS) 20°-42°**	@MN-UMALS- RET	@ SD -UMALS- RET	@MN-UMALS- NRET	@ SD -UMALS- NRET
**Lower Median Angle Light Scatter (LMALS) 10°-20°**	@MN-LMALS- RET	@ SD -LMALS- RET	@MN-LMALS- NRET	@ SD -LMALS- NRET
**Low Angle Light Scatter (LALS) 5.1^o**	@MN-LALS- RET	@ SD -LALS- RET	@MN-LALS- NRET	@ SD -LALS- NRET
**Axial Light Loss (ALL) A^o**	@MN-AL2- RET	@ SD-MN-AL2- RET	@MN-AL2- NRET	@ SD-MN-AL2- NRET
**MALS, (UMALS+LMALS)**	@MN-MALS- RET	@ SD -MALS- RET	@MN-MALS- NRET	@ SD -MALS- NRET

Footnote:

Abbreviations:

MN= Mean, SD= Standard Deviation, volume (V), conductivity (C), Axial Light Loss (ALL) A^o, Low Angle Light Scatter (LALS) 5.1^o, Lower Median Angle Light Scatter (LMALS) 10°-20°, Upper Median Angle Light Scatter (UMALS) 20°-42°, MALS, (UMALS+LMALS)

Example of interpretation of the table:

@MN-V-RET = Mean volume of reticulocyte

@SD-V- RET = Standard deviation volume of reticulocyte

@MN-MALS- RET = Mean of scatter angle MALS for reticulocyte

@SD-MALS- RET = Standard deviation of Mean of scatter angle MALS for reticulocyte

2. Microcytic Anaemia Factor (MAF) is a calculated formula available in Unicel DxH800 full blood count analyser by Beckman Coulter

MAF= (Haemoglobin*MCV)/100
